# Mistaken Identity: Misidentification of Other Vascular Structures as the Inferior Vena Cava and How to Avoid It

**DOI:** 10.3390/diagnostics14192218

**Published:** 2024-10-04

**Authors:** Josh Zimmerman, Candice Morrissey, Nibras Bughrara, Yuriy S. Bronshteyn

**Affiliations:** 1 Department of Anesthesiology, University of Utah School of Medicine, Salt Lake City, UT 84132, USA; 2 George E. Wahlen VA Medical Center, Salt Lake City, UT 84148, USA; 3 Department of Anesthesiology, Albany Medical Center, Albany, NY 12208, USA; 4 Department of Anesthesiology, Duke University School of Medicine, Durham, NC 27710, USA; 5 Durham Veterans Health Administration, Durham, NC 27705, USA

**Keywords:** ultrasound, cardiac ultrasound, point-of-care ultrasound, POCUS, echocardiography, inferior vena cava, IVC, hepatic veins, right hepatic vein, abdominal aorta

## Abstract

While point-of-care ultrasound (POCUS) of the inferior vena cava (IVC) is broadly perceived as having value in intravascular volume status assessment, this has not been borne out in large-scale meta-analyses containing heterogenous populations of acutely ill patients. While the limitations of IVC POCUS could be largely due to the complexity of the relationship between IVC appearance and volume status, another confounder not widely appreciated is the ease with which the aorta or right hepatic vein (RHV) can be mistaken for the IVC. While misidentification of the aorta as the IVC has been recognized elsewhere, misidentification of the RHV for the IVC has not and, in our experience, occurs frequently, even in the hands of experienced sonographers. We demonstrate how these errors occur and provide guidance on how to systematically avoid them.

Fundamental to the care of acutely ill patients is the accurate assessment of intravascular volume status. However, intravascular volume assessment by physical exam is often unable to detect clinically significant cases of hypo- and hypervolemia [1,2,3,4]. Further, other bedside intravascular volume assessment tools such as central venous pressure and pulse pressure variation also have severe limitations [5,6,7]. Thus, the search continues for adjunctive volume status assessment tools that are portable, inexpensive, non-invasive, and accurate.

Toward this goal, point-of-care ultrasound (POCUS) of the inferior vena cava (IVC) has been evaluated as a tool to estimate right heart preload in a growing number of clinical contexts [8,9,10]. However, in aggregate, these studies have shown that both IVC size and respirophasic change are also poor predictors of volume-recruitable cardiac output [11,12,13,14,15]. While some of the limitations of IVC POCUS could be due to the inherent complexity of the relationship between IVC appearance and right heart preload, another contributor to the heterogeneity of data may be an error that we observe commonly in clinical practice: misidentification of either the aorta or the right hepatic vein (RHV) as the IVC.

Misidentification of the aorta as the IVC has, to our knowledge, only been described once in the peer-reviewed literature [8]. However, we observe it commonly in clinical practice. In our collective experience of teaching diagnostic POCUS to hundreds of medical trainees and over-reading thousands of trainee-performed cardiac POCUS exams, we have observed this error frequently when trainees identify the IVC by relying on only a single criterion: that the IVC is the vascular structure that drains into the right atrium (RA). The problem with identifying the IVC this way is illustrated in Figure 1, which shows a long-axis view of the abdominal aorta that has been mislabeled by an ultrasound learner as the “IVC”. As shown in Figure 1, the cranial portion of the abdominal aorta can appear confluent with either the right atrium or another cardiac chamber. Further, we observe that while this problematic “RA-centric” method of identifying the IVC is widespread in colloquial ultrasound teaching (e.g., at point-of-care ultrasound workshops), the method is not supported by any cardiac ultrasound guidelines that we are aware of [10,16]. Instead, cardiac ultrasound guidelines merely acknowledge the presence of the RA in the IVC long-axis view but do not stipulate that the visualization of the RA is what reliably distinguishes the IVC from the aorta on ultrasound [10,16].

Instead, the IVC and abdominal aorta can be differentiated from one another in several ways. At the bedside, there are at least two reasonable approaches. First, one can place the ultrasound probe in the subxiphoid region in the midsagittal plane and fan the ultrasound beam slightly toward the patient’s left to obtain a long-axis view of the abdominal aorta (Figure 1) [17]. After establishing the aorta’s long-axis appearance in a given patient, one can then fan the ultrasound beam slightly towards the patient’s right side to attempt to visualize the IVC in long-axis. A second bedside approach is to begin with a short-axis view of both vessels (Figure 2) [8]. This IVC short-axis (SAX) view provides several helpful data points [18,19]. First, the IVC SAX view shows the visual differences between the IVC and aorta side-by-side for a given patient (e.g., each vessel’s depth, variability in size, pulsatility, wall thickness, location in relation to the liver, and motion in the body’s coronal plane). Second, the IVC SAX view helps to differentiate true IVC collapsibility from pseudo-collapsibility (i.e., movement of the transducer and IVC relative to one another in the body’s coronal plane during the respiratory cycle). Third, the IVC SAX view helps to locate the IVC in the abdomen to facilitate obtaining the long-axis view. I.e., once the IVC is found in the short-axis view, one can then center on the vessel and rotate 90 degrees counter-clockwise to obtain an IVC long-axis view.

Away from the bedside, determining whether an archived long-axis clip of an intra-abdominal great vessel was of the aorta or IVC is best performed by considering multiple criteria. Specifically, the abdominal aorta should have the following features: (1) lies posterior to the liver (never intrahepatic); (2) has thick echogenic walls; (3) has pulsatility; and (4) lacks respirophasic change in size [8]. In contrast, the IVC has the following features: (1) lies intrahepatically (see Figure 2B); (2) has thin walls; (3) only rarely has pulsatility (e.g., in cases of significant tricuspid regurgitation); and (4) can have significant respirophasic change in size [8]. Notably, when reviewing archived ultrasound clips, using only a single criterion to differentiate the aorta and IVC can be challenging, so it is prudent to consider all four of these factors rather than any of them in isolation [8].

Although misidentification of the aorta as the IVC remains an important and underappreciated problem in the practice of diagnostic POCUS, this problem has previously been briefly acknowledged in the peer-reviewed literature [8]. However, to our knowledge, misidentification of the RHV has not been previously reported. Further, in our collective experience over-reading thousands of POCUS exams at three academic medical centers, we observed that the misidentification of the RHV as the IVC occurs frequently, even in the hands of experienced sonographers. This error is potentially significant because the RHV is, in general, about 50% smaller in caliber than the IVC, so the misidentification of the RHV as the IVC is likely to cause underestimation of the patient’s preload [20].

As shown in Figure 3, when searching for the long-axis view of the IVC, it is easy to arrive at a long-axis view of the RHV inadvertently. This view is obtained with the probe only “partially rotated”, with the indicator pointing towards the patient’s left shoulder rather than toward the head (Figure 3C,D; Supplementary Video S2). To avoid this case of mistaken identity, it is helpful to use a systematic approach that includes identifying each of the following: IVC and aorta in the short axis (indicator toward the patient’s left; Figure 2), hepatic veins (indicator toward the patient’s left shoulder; Figure 3C,D), and long-axis views of the aorta (Figure 1) and IVC (Figure 3A,B).

Notably, several features of the IVC are also shared by the RHV. Both vessels (1) are intrahepatic; (2) contain thin walls; (3) have triphasic venous flow; (4) connect with the left hepatic vein (LHV); and (5) can appear to drain into the RA. So to distinguish the RHV from the IVC, additional characteristics need to be considered. In a sagittal view, the IVC traverses the center of the liver (toward 10 o’clock on the screen; Figure 1A), while the RHV originates from the right lobe of the liver (toward 8 o’clock on the screen; Figure 3C). The RHV makes an obtuse (>90 degree) angle with the LHV, while the IVC makes an acute angle (<90 degrees). The IVC will travel through the liver with no divisions, while multiple vessels may drain into the RHV. Finally, if the vessel appears small and non-collapsible, it should raise the suspicion that it is not the IVC.

## Figures and Tables

**Figure 1:**
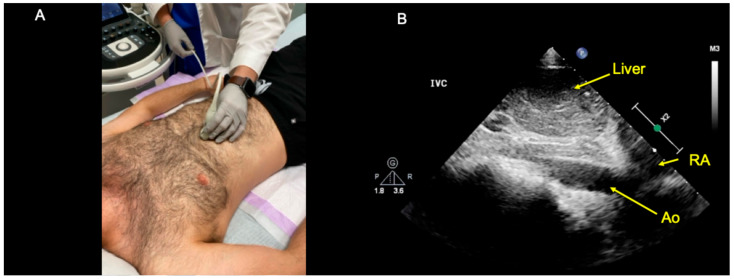
(**A**) Probe positioning used to obtain the subxiphoid long-axis view of the abdominal aorta. A low-frequency transducer is placed in the subxyphoid region in the parasagittal plane near the patient’s midline, with the probe indicator pointing cranially. The ultrasound beam is then fanned to the patient’s left side slightly until the aorta is visualized. (**B**) Example of a long-axis view of the abdominal aorta that was misidentified as the IVC by an ultrasound learner (the learner’s image label “IVC” remains included). As shown in this image, in a long-axis view of the abdominal aorta, the cranial portion of the aorta often appears confluent with the right atrium (RA) or other cardiac chambers. See also Supplementary Video S1 for additional examples of subxiphoid abdominal aorta long-axis views that ultrasound trainees have misidentified as the abdominal aorta when using a single criterion to identify the IVC (“the vessel that drains into the RA”).

**Figure 2:**
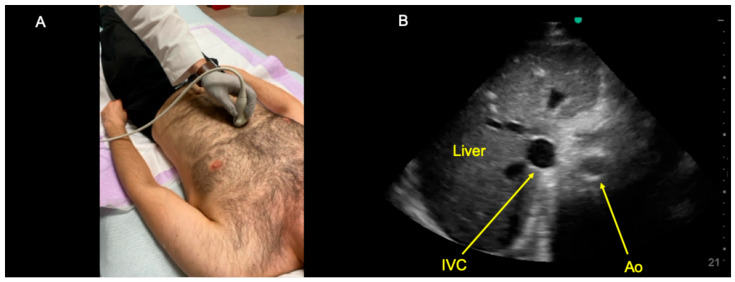
(**A**) Probe positioning used to obtain the subxiphoid short-axis view of both the inferior vena cava (IVC) and abdominal aorta (Ao). A low-frequency transducer is placed in the subxyphoid region in the transverse plane at the patient’s midline, with the probe indicator pointing toward the patient’s left side. The ultrasound probe is placed approximately perpendicular to the patient’s skin, and the beam is fanned cranially or caudally until the IVC and aorta are visualized in the short axis as shown in the right panel. (**B**) Example of a short-axis view of the abdominal aorta and IVC. As shown in this image, this short-axis view of both vessels provides useful data above and beyond what the long-axis view is capable of showing. Specifically, the short-axis view of these two great vessels (1) allows one to appreciate the visual differences between the two vessels in each individual patient; (2) permits recognition of lateral motion of the IVC relative to the transducer (a phenomenon that is difficult to distinguish from true IVC collapsibility when viewed solely from the IVC long-axis view); and (3) immediately reveals the location of the IVC in any given patient, facilitating the acquisition of the IVC long-axis view.

**Figure 3:**
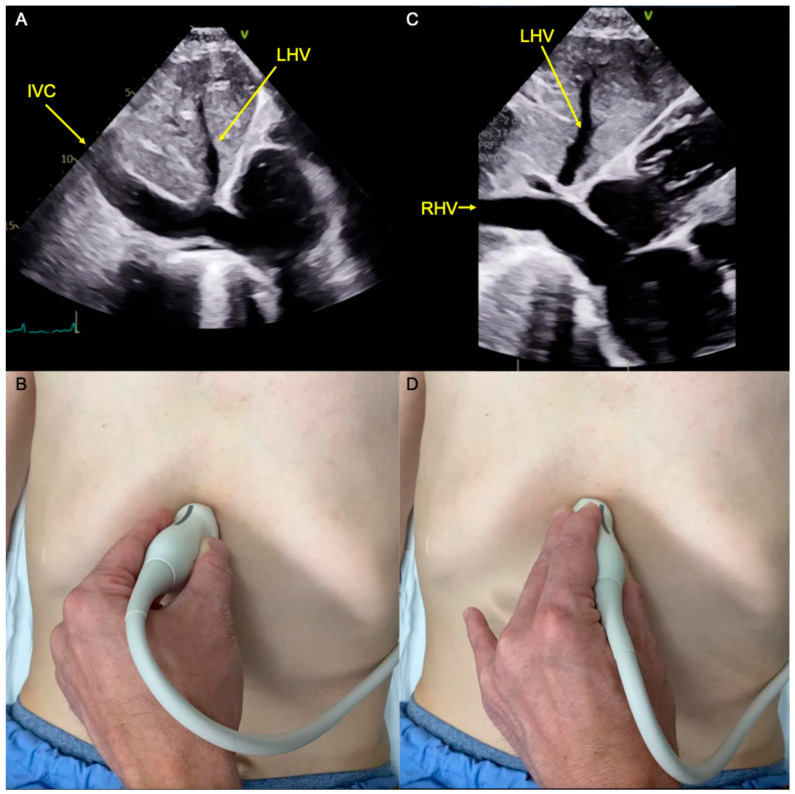
(**A**) Inferior vena cava (IVC) entering the screen at about 10 o’clock and coursing through the liver before draining into the right atrium. The left hepatic vein (LHV) is seen draining into the IVC at an acute (<90 degree) angle. (**B**) Demonstration of how Figure 1A was obtained by placing a low-frequency ultrasound probe in the subxiphoid region and aligning the ultrasound beam with the body’s sagittal plane such that the probe indicator pointing is toward the patient’s head. (**C**) Right hepatic vein (RHV) and left hepatic vein (LHV) making an obtuse (>90 degree) angle as they merge at the hepatic vein confluence. (**D**) Demonstration of how (**C**) was obtained by placing the ultrasound probe in an oblique plane such that the probe indicator is pointing approximately toward the patient’s left shoulder.

## Data Availability

De-identified ultrasound clips are included as Supplementary Videos.

## Supplementary Materials

The following supporting information can be downloaded at: https://www.mdpi.com/article/10.3390/diagnostics14192218/s1: Supplementary Video S1: Four examples of subxiphoid long-axis views of the abdominal aorta misidentified as the IVC by learners undergoing ultrasound training. In all cases, the cranial portion of the abdominal aorta appears confluent with either the right atrium or an ill-defined cardiac chamber. Supplementary Video S2: with the ultrasound probe placed in the subxiphoid region, video showing synchronous probe movement and ultrasound screen findings when rotating the probe from (a) the transverse plane where the IVC is seen in the short axis to (b) the oblique plane where the RHV is visualized in the long axis and then to (c) the parasagittal plane where the IVC and aorta can each be visualized in the long axis individually by fanning the probe from the right to left. (For additional examples, please see Zimmerman [21]).
